# hiPSCs Derived Cardiac Cells for Drug and Toxicity Screening and Disease Modeling: What Micro- Electrode-Array Analyses Can Tell Us

**DOI:** 10.3390/cells8111331

**Published:** 2019-10-28

**Authors:** Sophie Kussauer, Robert David, Heiko Lemcke

**Affiliations:** Department Cardiac Surgery, Medical Center, University of Rostock, 18057 Rostock, Germany; Sophie.Kussauer@med.uni-rostock.de (S.K.); Heiko.Lemcke@med.uni-rostock.de (H.L.)

**Keywords:** cardiomyocytes, multi-electrode-array, micro-electrode-array, MEA, drug/toxicity screening, field potential

## Abstract

Human induced pluripotent stem cell (iPSC)-derived cardiomyocytes (CM) have been intensively used in drug development and disease modeling. Since iPSC-cardiomyocyte (CM) was first generated, their characterization has become a major focus of research. Multi-/micro-electrode array (MEA) systems provide a non-invasive user-friendly platform for detailed electrophysiological analysis of iPSC cardiomyocytes including drug testing to identify potential targets and the assessment of proarrhythmic risk. Here, we provide a systematical overview about the physiological and technical background of micro-electrode array measurements of iPSC-CM. We introduce the similarities and differences between action- and field potential and the advantages and drawbacks of MEA technology. In addition, we present current studies focusing on proarrhythmic side effects of novel and established compounds combining MEA systems and iPSC-CM. MEA technology will help to open a new gateway for novel therapies in cardiovascular diseases while reducing animal experiments at the same time.

## 1. Introduction

The first generation of induced pluripotent stem cells (iPSCs) by Yamanka and co-workers in 2006 was a milestone for stem cell research as it allows the in vitro production of human cells without ethical concerns. Like embryonic stem cells, iPSCs have the capability to differentiate into any cell type, including cardiomyocytes, therefore providing an easy accessible cellular source for the generation of cardiac organoids and tissue structures [1,2,3].

One possible application for iPSC-derived cardiomyocytes (CMs) is their use in cell therapy replacing damaged tissue by in vitro generated CMs. As cardiovascular diseases are the major cause of death worldwide such regenerative approaches are needed for the development of novel treatment options. The potential and feasibility of iPSC-CM transplantation has been investigated in small and large animal models [4,5,6,7].

Thereby, an important future option of iPSC-CMs will be their generation from patient specific tissue enabling the implementation of autologous cell transplantation strategies. In this respect, iPSC-CMs can be used for the development of personalized drug screening approaches and clinically relevant diseases models. Therefore, iPSCs enable cost-effective methods to identify potential drug targets, even more accurately than animal models or other in vitro cell systems. Successful pre-clinical application of iPSC-derived cardiomyocytes for drug screening assays has been lately demonstrated by the CiPA initiative, which was initiated to assess the proarrhythmic risk of novel cardio therapeutics. A myriad of studies investigated in vitro drug effects on different ion channels of iPSC-CMs [8,9,10,11], reflecting the importance of electrophysiological measurements using stem cell derived cardiac cells. 

However, the maturation of iPSC derived CMs is still a critical point for their application in cardiovascular research as well as for clinical applications. Besides metabolic and structural maturation, proper ion channel composition is crucial for the development of a mature cardiac phenotype. During the last decade, extensive analyses have been performed on the electrophysiological properties of iPSC-CMs [12,13,14,15]. Several ion channels and ion currents have been found to be present in iPSC-CMs, including sodium (I_Na_), potassium (I_K1_ and I_Kr_), L-type and T-type calcium channels, etc. Although multiple differentiation protocols have been developed, researchers failed to generate fully mature cardiomyocytes in vitro, possessing identical electrophysiological properties as their native adult counterparts [16,17,18]. Moreover, it has largely been shown that iPSC-CMs represent a heterogeneous population of electrophysiological phenotypes, i.e., atrial, ventricular and nodal-like cells [19], each characterized by a specific electrical profile. Therefore, it is important to obtain electrophysiological data for detailed characterization of iPSC-CMs, in particular when differentiation into a certain cardiac subtype is desired [20,21].

Typical approaches to investigate the electrophysiological properties of stem cell derived CMs will be discussed in the following paragraph.

## 2. Methods for Electrophysiological Characterization of iPSC-CMs

Several different techniques exist to study the electrophysiological properties of cardiac cells, including patch clamp analysis, MEA measurement and fluorescence dye-based assessment of the membrane potential. Each of these techniques has its own advantages and limitations, which are described in detail in the following.

### 2.1. Patch Clamping

Patch clamping is the gold standard technique for the acquisition of ion current data and detailed measurement of action potential (AP) properties in individual cells. The basic principle of patch clamp relies on a blunt ended glass pipette that is sealed onto the cellular membrane to obtain a so-called gigaseal [22].

In the “current patch clamp” mode the membrane potential is recorded while the current applied by the patch pipette is controlled by the operator [23]. The current patch clamp technique allows detection of APs that occur spontaneously or after stimulation with a current change induced by the recording pipette. Considering the fact that iPSC-CMs also contain non-beating populations, current patch clamp methodology allows the detection of AP patterns in these quiescent cells [23,24]. Moreover, detailed AP features, such as AP duration, amplitude, beating rate and mean diastolic potential can reliably be acquired with current patch clamping [25].

When precise characterization of ion channel subtypes is desired, “voltage patch clamp” is performed to measure individual ion currents. Unlike in the current patch clamp mode, the operator keeps the membrane potential at a certain value that enables detection of the net membrane current. In hiPSC-CMs, voltage patch clamp has been successfully applied to obtain data about ion channel density, voltage dependency and activation/deactivation characteristics [23]. 

However, these manual patch clamp methods are complex, technically challenging procedures that require high operator skills as well as a biophysical background for data interpretation. Another limitation is the low throughput since measurements are usually performed on the single cell level. Therefore, automated patch clamp devices have been developed to overcome the aforementioned drawbacks of manual patch clamp approaches [26]. Automatic platforms profoundly increase the efficiency of electrophysiological data recording by assessment of 10–700 cells at the same time [27]. However, while automated systems are capable to analyze hundreds of cells under variable experimental conditions, the accuracy of obtained data is reduced if compared to manual patch clamping [28,29]. High-throughput analysis is realized by analysis of single cell suspensions, in contrast to manual patch clamping where cells are usually processed in an adherent state. Recently developed systems are equipped with temperature control, optical stimulation and internal perfusion systems to ensure high data quality and reproducibility [27,30]. Data consistency and robustness is further determined by the homogeneity and density of the applied single cell suspension—a point that is particularly important for iPSC-CMs that are sensitive to dissociation as it can affect membrane proteins and electrical physiology of the cell, including ion channel expression [31,32]. Automatic techniques also do not provide the possibility of selective cell capturing. Hence, the system demands highly purified cell populations, which could be challenging when working with CMs differentiated from iPSCs that commonly represent a mixture of different cardiac subtypes [29]. 

### 2.2. Optical Recordings of the Membrane Potential 

An indirect technique to assess electrophysiological data of iPSC-CMs is the application of voltage-sensitive dyes that change fluorescence intensity or emission spectra upon alteration of the membrane potential. Considering fluorescence microscopy as one of the most commonly used methods in cell research, utilizing voltage-sensitive dyes is operationally simple and does not require special instrumentation. Moreover, it is less invasive and it enables monitoring of voltage dynamics over thousands of cells with very high temporal resolution [33]. Several studies have proven feasibility of voltage sensor probes for drug screening experiments in iPSC-CMs [34,35,36]. Recently, Takaki and colleagues [37] applied voltage sensitive dyes for the identification of distinct cardiac subtypes in an iPSC-CM population. Further, the authors were able to detect differences in the AP pattern in iPSC-CMs obtained from patients suffering from the long QT syndrome, compared to control cells.

Alternatively, voltage sensitive probes can be engineered as fluorescent proteins that are stably expressed in target cells. Compared to voltage-sensitive dyes, these proteins possess lower phototoxicity, thus, facilitating long-term measurements. These genetically encoded probes are designed by conjugating a voltage-sensing domain to a single fluorescent protein, a fluorescence resonance energy transfer (FRET) pair or rhodopsin proteins [38,39]. Changes of the membrane potential induce conformational rearrangement of the voltage-sensor, which in turn modulates the emission spectra of the attached fluorescent protein. The latest generation of genetically encoded voltage sensors, such as ArcLight, Archer1 or QuasAr1, show large fluorescence alteration upon depolarizing events (40%–80% for a 100 mV depolarization) associated with faster on/off kinetics (1–10 ms). Shaheen et al. generated ArcLight expressing human iPSC-CMs to establish a 2D cardiac tissue platform for optical mapping and pharmacological studies [40]. Former data confirmed the suitability of genetically encoded voltage sensors for iPSC-CM drug screening applications and disease modeling attributed to altered AP phenotypes [41,42,43,44]. However, there are certain limitations of this technology. Like fluorescence dyes, genetically encoded voltage indicators provide only relative, not absolute values for the membrane potential [45]. The lower on/off kinetics increase the probability of losing high frequency AP elements [41,45]. Furthermore, introduction of voltage-sensitive proteins like ArcLight could affect the electrophysiological properties of iPSC-CMs. This needs to be carefully addressed by the operator as well as proper folding and membrane integration of the voltage sensitive probe.

### 2.3. MEA-Based Analysis of Cell Behavior 

**Figure 1:**
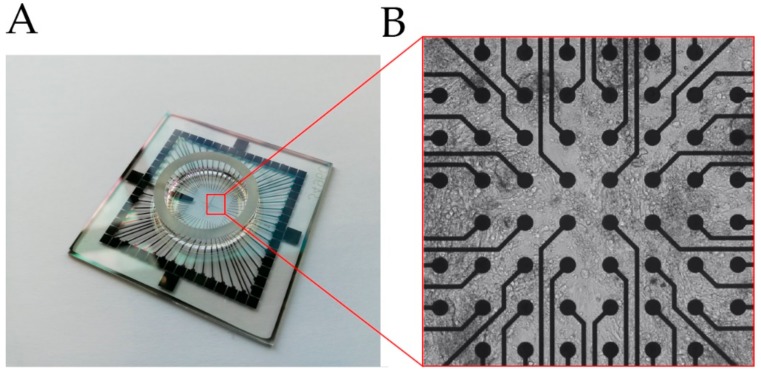
(**A**) Glass multi-/micro-electrode array (MEA) chip used to detect field potential (FP) of cells. (**B**) Cells seeded on an MEA surface, grown on top of the electrodes (black dots), Video S1.

A common MEA system is composed of dot-like electrodes arranged in two-dimensional grids that measure the fluctuations in the extracellular field potential (FP) of an attached cell layer in respect to a reference electrode placed outside the grid (Figure 1). MEA is a non-invasive, label free methodology that has been initially applied to investigate neuronal activity [46]. However, in recent years an increasing number of studies have taken advantage of MEAs to particularly analyze compound-induced cardiac toxicity in iPSC-CMs [47,48,49]. Like optical recordings of the membrane potential, MEA systems allow non-invasive and cost-effective measurements at high throughput scale, and long-term observations [46,50]. On the other hand, Rynnännen et al. published data of a custom-made MEA platform for FP detection based on a single cell analysis [51]. In contrast to conventional MEA systems, this optimized device demonstrated a modified layout of larger electrodes, most suitable for observation of single iPSC-CMs. Similarly, agarose micro-chambers printed on MEA have been found to facilitate single cell detection of FPs in stem cell-derived CMs [52]. Moreover, electrophysiological assessment using MEAs is not only restricted to cell culture but can also be performed on the tissue level to better simulate in vivo conditions, as shown for murine and human heart tissue slices [53,54,55].

An advantage of the MEA technology is its high flexibility as it can be combined with other detection methodologies to multiply the number of parameters describing cellular functions. The main parameter assessed is the FP of spontaneously beating CMs that can be correlated with certain elements of the AP pattern. Additionally, newly developed platforms provide the possibility to detect impedance of the attached cell layer [56,57]. Unlike the FP that reflects the electrical activity, impedance corresponds to the mechanical movement of the cell on the electrode. It is influenced by cell density, cell number and the extent of cell adhesion. Thus, measuring impedance helps to acquire valuable information about beating behavior, proliferation, cell death and viability [22,58].

A relationship between contraction parameters and electrophysiological activity has also been investigated by combination of MEA and high-speed video microscopy, followed by motion based image analysis of beating cells [59]. Likewise, fluorescence microscopy was used to correlate FP measurements with subcellular information [60]. However, the combined setup of MEA platforms with optical techniques requires certain structural features to achieve optimal visualization of target cells, such as transparent electrodes [51,60,61].

In another study, Siemenov et al. developed a combined scanning ion conductance microscopy–MEA system for simultaneous detection of cell surface morphology and FP in cardiomyocytes [62]. The platform reveals morpho-dynamic parameters, including maximum displacement and cell volume changes in a time-dependent manner. Together with the FP data obtained from MEA measurements, the authors were able to reconstruct 3-dimensional motion of the cell surface over a complete contraction-relaxation cycle [62].

In order to obtain reproducible and reliable experimental data, a number of points need to be considered when working with MEA systems that are particularly important for drug screening assays. Since individual iPSC-CMs show variations in AP waveforms [63], confluent monolayer cell sheets are preferred to reduce the variability of the acquired FP patterns. In this regard, cell density needs to be carefully addressed by the operator as it was found to influence electrical remodeling of CMs derived from human iPSCs [64].

## 3. Action Potential vs. Field Potential

Both, AP and FP are parameters describing the membrane potential of cardiomyocytes or any other cell type that is electrically active. They are generated by ion currents between the extra- and intracellular space, tightly regulated by several different membrane-located ion channels [65]. In drug development, pharmacological compounds are classified in respect to their cardiotoxic effects based on the AP or/and FP pattern in iPSC-derived CMs [34]. In addition, electrophysiology is used to identify and characterize the different cardiac subtypes in iPSC derived CM populations, which is crucial for specific cell programming strategies [20,66,67].

### 3.1. Action Potential in Native Cardiac Cells and iPSC Derived CMs

The AP represents the time-dependent alterations of the membrane potential in CMs that occur during the contraction of heart tissue. This requires a well-defined orchestration of numerous ion channels. Since the human heart comprises different cardiomyocyte subtypes, the AP pattern varies significantly, depending on the regions of heart (e.g., atrium, sinus node and ventricle) [68].

**Figure 2:**
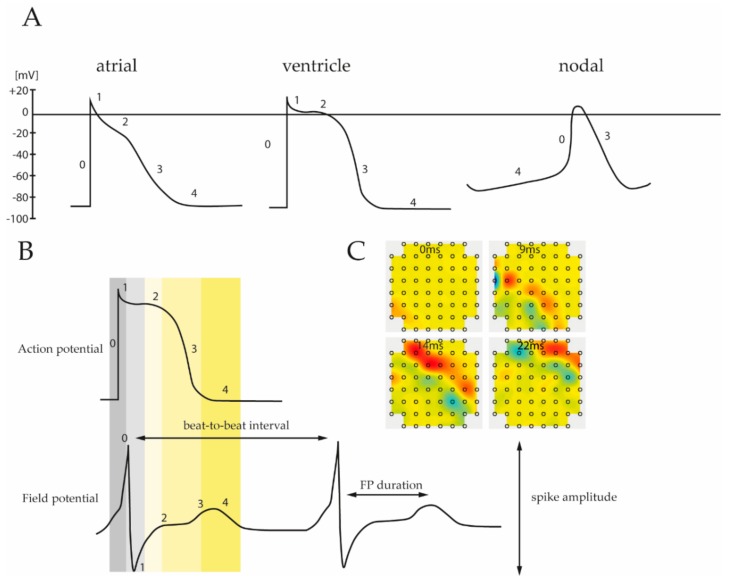
(**A**) Subtype specific pattern of the cardiac action potential. Ventricular, atrial and nodal cells are characterized by unique depolarization and repolarization processes leading to different action potential (AP) waveforms. Numbers correspond to the different phases that reflect the activity of involved ion channels. (**B**) Comparison of the different phases between recorded action potential and field potential. As field potential measurements allow reconstruction of the corresponding action potential it provides important physiological parameters of electrically active cells, including spike amplitude, FP interval, etc. (**C**) Moreover, MEA analysis can be applied to obtain data about prolongation velocity and direction of the field potential spreading throughout the cell layer.

Despite this electrical heterogeneity, each subtype specific pattern consists of five different phases, reflecting the activity of certain ion channels (Figure 2A). Based on an incoming depolarization stimulus, opening of voltage-gated Na^+^ channels induces sodium influx into the cytoplasm, resulting in a rapid depolarization of the membrane potential up to +20 to +40 mV (Phase 0). Subsequently, phase 1 is determined by time and voltage-dependent opening and closing of various ion transporters permeable to Na^+^, Ca^2+^ and K^+^, leading to a slight, transient hyperpolarization of the membrane potential (−10 to −30 mV). The following phase 2 is characterized by a relatively high capacity of the cell membrane and it is primarily driven by depolarization-dependent L-type Ca^2+^ channels. Due to a balanced interplay between inward currents of calcium and efflux of potassium phase 2 demonstrates a plateau period, which is particularly prominent in ventricular muscle cells [68,69]. During the plateau phase, Ca^2+^ channel conductance decreases while the outward current of K^+^ inclines. This in turn promotes further repolarization (phase 3) leading to a resting potential of ~−85 mV (phase 4). 

As stated above, AP patterns are unique for each cardiac subtype resulting from different ion channel composition within the cellular membrane (Figure 2A). Nodal cells, found in sinoatrial and an atrioventricular AV node or His-bundles, are capable to generate their own AP without an additional depolarizing stimulus. Compared to atrial and ventricular cells, the resting potential in nodal cells is unstable, begins at ~−60 mV (vs. ~−85 mV in atrial and ventricular cells) and gradually increases towards a threshold. This “pacemaker potential” is generated by K^+^ channels that open slowly upon depolarization and deactivates with time. Concurrently, depolarization of about −60 mV activates a nodal specific Na^+^ channel, known as the “funny channel”, causing an increase of the intracellular Na^+^ level. Once a threshold is reached, opening of voltage-gated Ca^2+^ channels induce a strong upstroke. This is in contrast to atrial and ventricular cells where a Na^+^ influx mainly contributes to the rapid depolarization in phase 0.

Differences in the AP pattern are also distinct between atrial and ventricular regions of the heart. Atrial CMs undergo a more rapid early repolarization and demonstrate a less profound plateau phase, followed by slow phase 3 repolarization (Figure 2A). These differences emanate from specific K^+^ channels, expressed in atrial cells, but not found in ventricular tissue [68].

Analysis and classification of AP patterns of iPSC-CMs is challenging as they demonstrate a large amount of variability, which supports the notion that CMs derived from iPSCs are a mixture of different cardiac subtypes of distinct maturation level [16,19,70]. Although multiple differentiation protocols have been established, researchers failed to generate fully mature cardiomyocytes in vitro possessing identical electrophysiological properties as their native adult counterparts [16,18]. For example, Ronaldson-Bouchard et al. have shown structural and metabolic maturity and adult-state like gene-expression of three iPSC cell lines after cultivation as cardiac tissues for four weeks, it remains to be seen whether this approach can be generally transferred to any iPSC cell line [71]. In addition, it is difficult to compare APs among different studies because of various experimental conditions used. Nevertheless, a common feature of iPSC-CMs compared to native CMs is their ability to generate APs without the need of an external, depolarizing signal, indicating a relative similarity with nodal tissue cells. Indeed, iPSC-CMs were found to express funny channels to drive spontaneous activity. Another nodal-cell characteristic of iPSC-CMs is their relatively positive resting potential of −60 to −70 mV that mainly relies on a lower or absent expression of the K^+^ channel I_K1_ [16,72], allowing the depolarizing funny current to trigger the AP. This low level of I_K1_ further evokes a slower upstroke velocity in phase 0. Computational simulations revealed that an increasing expression of I_K1_ in iPSC-CMs would induce a more negative and more stable resting potential [16,72,73,74]. These data also indicate an immature state of iPSC derived-CMs and suggests possible limitations for cardiovascular research and clinical applications.

### 3.2. Field Potential

Classically, the cardiac action potential of single cells is analyzed using patch clamp devices, which allow detection of each individual ion current contributing to the AP pattern [69,75]. In contrast, MEA does not directly measure the AP but rather record cardiac FP instead, shown in Figure 2B. The FP encompasses the spatiotemporal electrical activity of cell clusters attached to the electrode, thus, it is the superposition of all ionic processes, ranging from fast action potentials to slowest fluctuations [76,77]. The measured FP arises from spreading of the cardiac AP throughout the cell monolayer relative to the recording electrodes. Therefore, it is comparable to the clinical electrocardiogram signal that represents voltage change over time due to electrical activity of the heart [47].

Since the biophysical processes underlying the generation of FPs are well known, it is possible to reconstruct the corresponding AP pattern and to extract important physiological parameters [76]. Figure 2B depicts the different phases of a typical ventricular AP pattern and the corresponding FP measured by MEA. The FP waveform contains a strong transient spike attributed to the Na^+^ influx and associated membrane depolarization, followed by a gentle incline based on the intracellular increase of Ca^2+^ level and ending with repolarization associated with K^+^ efflux. In addition, a comparative analysis of patch clamp data and MEA recordings revealed that duration of the FPs correlate well with the length of the QT interval of APs [78]. Similar results were obtained by Asakura et al., showing that MEA-based FP detection can be applied to determine the prolongation of the QT interval following drug administration in iPSC-derived CMs [49]. In addition to the QT interval and K^+^/Ca^+^ flux, the FP pattern provides valuable information about the beating frequency as well as AP duration (Table 1, Figure 2B). Moreover, since MEA measurements are commonly performed on cell monolayers, propagation and direction of the AP can be determined (Figure 2C, Video S2).

For a more precise comparison of FPs and patch clamp measurements, MEA platforms have been developed that allow the detection of APs. These local extracellular AP assays, utilize electrodes capable to apply electrical stimulation in order to induce small pores in the cellular membrane for the acquisition of stable AP patterns over longer timescales [79,80]. In addition, the use of 3-dimensional electrodes can facilitate the coupling intensity and decrease the membrane resistance of individual cells required for intracellular recordings [81,82].

## 4. Application of MEAs for Cardiotoxic Risk Assessment

In 2014 the US department of health and human services estimated that nearly 1 million patients show adverse drug reactions each year—among these drug induced arrhythmias are the leading cause [83].

The comprehensive in vitro proarrhythmia assay (CiPA) initiative was originated for drug proarrhythmic potential assessment in order to analyze several known drugs and substances on their potential to affect the cardiac system. Thus, a list of 28 relevant drugs with a potential effect was published. The list reaches from vandatanib, clarithromycin, droperidol over metoprolol to tamoxifen and verapamil to name only a few. The drugs were categorized into high risk, intermediate risk and no or very low risk for torsade-de-pointes-tachycardia (TdP; Table 2). TdP is characterized by polymorphic ventricular tachyarrhythmias, which can follow drug induced delayed ventricular repolarization (OT interval prolongation) [84,85]. For the categorization the initiative recommends and describes assays that are mechanistically based in vitro assays and are composed of four different steps that in total should give a comprehensive overview of the possible proarrhythmic potential: 

First, the effect of potential drugs and substances on several cardiac ion currents, which are defined as a core set of ion channel types needs to be analyzed. Second, the electrophysiological properties are simulated in in silico models. Since ventricular cardiomyocytes can be generated from human stem cells they represent a promising platform for drug testing, consequently the drug effects are measured in this in vitro setting as the third step. Finally, the expected and unexpected effects on the entire human organism need to be clinically evaluated [84].

Since the inception of the CiPA initiative in 2013, the analysis of the listed drugs was set into focus of research by the research community. Several cell lines (including self-generated and commercially available cell lines like iCell Cardiomyocytes (Fuji), Pluricytes (Pluriomics), Cor4u (Ncardia), Axol Bioscience, i-HCm (Cell applications), ASC (Applied Stem Cell, ix Cells Biotechnologies), CDI (Cellular Dynamics International), Cellartis (Clontech, Takara), ReproCardio (ReproCELL) and ACCEGEN (immortalized from patients or transdifferentiated from hSC) have been used to analyze the impact of compound administration on cardiomyocyte electrophysiology. The methodology used for the measurements range from (automated) patch clamp over MEA to optical measurement. 

There is a tendency noticeable promoting the assessment of not only hERG inhibition or QT prolongation but also analysis of Nav1.5 (voltage gated Na^+^ channel), Cav1.2 (voltage gated Ca^2+^ channel) or of index of cardiac electrophysiological balance (assesses balance between OT interval and QRS duration).

The Consortium for Safety Assessment using Human iPS cells (CSAHI) was established in 2013 by the Japan Pharmaceutical Manufacturers Association in order to “give recommendations for the usage of human iPS-cell derived cardiomyocytes, hepatocytes and neurons in drug testing evaluation” [87]. The CSAHI study is also aiming at the analysis of potential unknown effects on the cardiac system and tries to overcome/decrease proarrhythmic-risk market withdrawal. There have been several substances being tested that are not listed on the CiPA list. CSAHI provides a generalizable platform with the promising method for prediction of cardiotoxicity [88]. There are different parameters that are analyzed for the named prediction, such as QT prolongation, arrhythmia, but not only using the hERG assay. The latter is known to be an inaccurate predictor, because it only focuses on the inhibition of this particular ion channel—yet for cardiac adverse effects, mostly a broader range of (different) ion channels is affected [89].

Using animal hearts as a model, (which is also done for drug testing approaches) or the guinea pig papillary muscle action potential assay (qpAPD), is also not sufficient due to interspecies differences in electrophysiological properties and different responding behavior to drugs [87,90].

The tested substances are modulating a range of cardiac ion currents and consequently can have multiple arrhythmogenic effects. Due to this fact, multiple parameters are required to be evaluated, especially when using MEA technology. The electrophysiological response to drugs can be analyzed using the heart rate, field potential duration (FPD) and the corrected FPD (cFPD), all indicating arrhythmia-like waveforms [85]. Moreover, further analytical parameters are available using impedance measurement, deformation analysis or high content imaging [87].

The usage of human iPSC-CMs for drug testing is a promising tool due to their large-scale production circumventing the lack of a source for human adult cells [85]. However, they are displaying/carrying the disadvantage of not being identical to isolated primary adult cardiomyocytes and showing indicators for immature state, iPSC-CM are comparable in expression of cardiomyocyte marker. Especially the expression of relevant cardiac ion channels such as I_Na_, I_CaL_, I_f_, I_to_, I_K1_, I_Kr_ and I_Ks_ has been analyzed [91,92]. Compared to previously used single ionic current model approaches they have shown a higher sensitivity and specificity [85]. Techniques such as qpAPD evaluated several false negative results [87], leading to possibly high risk in drug administration on patients. Many attempts have been carried out to analyze the response of human iPSC-CM to the administration of not only cardiogenic drugs and to further compare it to human adult cardiomyocytes. Yet, limitations must be kept in mind when transferring results from cell models to the human organism.

Since comparability within CiPA associated data generation is crucial Kanda et al. aimed to develop a standardized protocol for the experimental data generation including experimental conditions and calibration compounds providing it to a big community [63].

In order to validate the reliability and comparability of iPSC-CM based drug testing CiPA associated studies have been examined using a batch of known (formerly analyzed) drugs, various commercial cell lines, different electrophysiological platforms and multiple experimental sites [85,93]. Differences between the various analyzed combinations could be seen but also representative effects on depolarization, confirming the utility of the CiPA paradigm. Promoting the concept of CiPA, the CSAHI study from Japan HEART TEAM could not detect any inter-facility variability [90] and is providing new insights from their large scale drug testing combining electrophysiological data (from the MEA platform) with gene expression profiles [87,88]. A comprehensive overview of tested substances on their proarrhythmic risk/cardiac side effects using the combination of MEA technology with iPSC-CM is given in Table 3 and Table 4. Table 3 summarizes drugs with a primarily non-cardiac medical indication such as antibiotic or antipsychotic drugs. Anti-arrhythmic drugs and cardiac ion-channel blocker are included in Table 4, containing cardiogenic substances.

To consider the influence of serum containing medium during administration and measurement of potential cardiotoxic drugs Schocken et al. compared serum containing and serum free medium in pro-arrhythmia risk assessment. The solubility of a drug connected with the precise drug concentration as well as cardiomyocyte electrophysiology may be affected by the serum. Mostly the precise serum composition is unknown. Using a high-throughput MEA 25 substances have been analyzed, showing differences in drug availability and the tendency of serum to influence the FPD in an increasing or decreasing manner for several drugs [94].

To further improve and expand the system of iPSC-CM drug testing, Zeng et al. addressed the diversity of iPSC-CM models from different gender and ethnical origin with known pharmaceuticals, detecting possible inter-sex differences [95]. Therefore, they prefer/vote for generalized pre-set acceptance criteria for iPSC-CMs. Burnett et al. recently published a study using not only a population-based CM model, generated from cells of 43 individuals (both gender and diverse ancestry) to defeat the drawback of inter-individual variability but also tested a large scale of substances of pharmaceuticals, environment and food. Both for control and substrate administration they found inter-individual variability, increasing the requirement of population based-models (to reproduce a whole population) [96].

**Table 3:** MEA based safety testing of drugs without cardiac indication using human induced pluripotent stem cell cardiomyocyte (hiPSC-CM).

Substance	(Site of) Action	Effect	Min. Effective Conc.	Cell Type/ Subtype	Differentiation Protocol	Age/ Maturation State	Platform	Reference
Alfuzosin	Treatment of benign prostatic enlargement, a hERG-channel blocker	Clinical QT prolongation	30 nM	hiPSC-CM iCell™ (mixture of ventricular, atrial, nodal cells)	n/a	32 days of differentiation +15–26 days	MEA	[87,88]
Astemizole	Antihistaminergic drug, H1 receptor antagonist, multi-channel block	Repolarization prolongation/arrhythmogenic effects, hERG channel blockade	3–10 nM	hiPSC-CM iCell™/iCell^2^™/Cor4U^®^ (mixture of ventricular, atrial, nodal cells)	n/a	32 days of differentiation (+15–26 days) n/a	MEA	[87,88,93]
BaCl_2_	Digitalis like activity, stimulation tonic contraction in muscle, used as contrast agent	Chronotropic effect K^+^ and Ca^2+^ modulation	-	hiPSC-CM iCell™ (mixture of ventricular, atrial, nodal cells)	n/a	32 days of differentiation +15–26 days	MEA	[87]
Blebbistatin	Myosin II ATPase inhibitor	Increase in beating frequency, beating arrest (30 µM)	1–30 µM	hiPSC-CM iCell™ (mixture of ventricular, atrial, nodal cells)	n/a	Min. 32 days of differentiation	MEA	[88]
Carbachol	Parasympathomimetic drug cholinergic agonist K_Ach-_ channel, glaucoma treatment	Negative chronotropic effects, FPDc prolongation, decrease in beating frequency	10 µM	Double reporter cell line, subtypes: ventricular, atrial, nodal, TBX5 Nkx2.5/hiPSC-CM (iCell™ mixture of ventricular, atrial, nodal cells)	2D n/a	35-40 day of differentiation 32 days of differentiation	Patch clamp, MEA	[21,88]
Chlorpromazine	Anti-psychotic drug, multi-channel block	Early afterdepolarization, beating arrest	10 µM	hiPSC-CM iCell™ mixture of ventricular, atrial, nodal cells)	n/a	32 days of differentiation	MEA	[88]
Chromanol 293B	IKv7.1 channel Blocker	Prolong FPD in control cells,		LQTS cells and control (patient- derived cells) n/a	3D	30-60 days of differentiation +50 days	MEA	[97]
Cisapride	Prokinetic gastrointestinal drug, multi-channel block	Prolongation of FPD, Repolarization delays/arrhythmogenic effects Prolongation of QT from patients with long QT syndrome	100 nM	hiPSC-CM Cor4U^®^ and iCell™ mixture of ventricular, atrial, nodal cells Wt iPSC, and from patient with LQTS (n/a)	n/a 3D	10 days after differentiation, min. 32 days of differentiation n/a	MEA, automated patch clamp	[26,87,88,93,98,99]
Clarithromycin	Antibiotic drug	Repolarization prolongation, arrhythmogenic effects	-	hiPSC-CM iCell^2^™/Cor4U^®^ (mixture of ventricular, atrial, nodal cells)	n/a	32 days of differentiation n/a	MEA/VSO	[93]
Clozapine	Anti-psychotic drug, multi-channel block	Shortening of FPDc, increase in beat frequency	0.3–1 µM	hiPSC-CM iCell™ mixture of ventricular, atrial, nodal cells)	n/a	32 days of differentiation	MEA	[88]
Domperidone	Dopamine-antagonist, anti-nausea drug hERG- channel blocker	Repolarization prolongation, arrhythmogenic effects	10 nM	hiPSC-CM iCell™/iCell^2^™/Cor4U^®^ mixture of ventricular, atrial, nodal cells)	n/a	32 days of differentiation n/a	MEA/VSO	[88,93]
Doxorubicin	anthracycline chemotherapy agent	Decrease in FPD, beat frequency and spike amplitude	1 µM	hiPSC-CMs iCell™ 50% ventricular, 10% atrial cells patient derived cells (n/a)	n/a 2D	32 days of differentiation 20–30 days of differentiation	MEA	[100,101]
Droperidol	Neuroleptic drug	Repolarization prolongation, arrhythmogenic effects	-	hiPSC-CM iCell^2^™/Cor4U^®^ (mixture of ventricular, atrial, nodal cells)	n/a	32 days of differentiation n/a	MEA/VSO	[93]
Fluoxetine	Anti-depressant drug	Clinical QT prolongation	-	hiPSC-CM iCell™ (mixture of ventricular, atrial, nodal cells)	n/a	32 days of differentiation +15–26 days	MEA	[87]
Isoproterenol	Bronchodilator	Chronotropic effect, K^+^ and Ca^2+^ Modulation, FPDc shortening, increasing beating frequency	3–100 nM	hiPSC-CM iCell™ (mixture of ventricular, atrial, nodal cells)	n/a	32 days of differentiation +15–26 days	MEA	[87,88]
Loratadine	Anti- histaminergic drug, H1 receptor block, multi-channel block	Increase in beating frequency	0.1–3 µM	hiPSC-CM iCell™ (mixture of ventricular, atrial, nodal cells)	n/a	32 days of differentiation	MEA	[88]
Moxifloxacin	Anti-biotic drug, multi-channel block	Repolarization delay	10 µM	hiPSC-CM iCell™/Cor4U^®^ (mixture of ventricular, atrial, nodal cells) GE Healthcare (Cytiva™), Stanford Cardiac Institute	n/a	32 days of differentiation +14–24 days n/a	MEA	[85]
Ondansetron	Antiemetic drug, serotonin-receptor block	Repolarization prolongation, arrhythmogenic effects	30 nM	hiPSC-CM iCell^2^ ™/Cor4U^®^ (mixture of ventricular, atrial, nodal cells)	n/a	32 days of differentiation n/a	MEA/VSO	[93]
Pimozide	Anti-psychotic drug, multi-channel block	Repolarization prolongation/arrhythmogenic effects	3–10 nM	hiPSC-CM iCell™/iCell^2^ ™/Cor4U^®^ (mixture of ventricular, atrial, nodal cells)	n/a	32 days of differentiation +15–26 days n/a	MEA	[87,93]
Risperidon	Anti-psychotic drug, serotonin-receptor block	Repolarization prolongation	3–30 nM	hiPSC-CM iCell^2^™/Cor4U^®^ (mixture of ventricular, atrial, nodal cells)	n/a	32 days of differentiation n/a	MEA	[93]
Sunitinib	Anti-cancer drug, tyrosine kinase inhibitor	FPDc prolongation, early afterdepolarization	0.3–10 µM	hiPSC-CM iCell™ (mixture of ventricular, atrial, nodal cells)	n/a	32 days of differentiation	MEA	[88]
Terfenadine	Anti-histaminergic drug, H1 receptor block	FPDc prolongation, decrease in spike amplitude, repolarization prolongation	100–1000 nM	hiPSC-CM iCell™/iCell^2^™/Cor4U^®^ (mixture of ventricular, atrial, nodal cells)	n/a	32 days of differentiation n/a	MEA	[93,98]
Tetrodotoxin (TTX)	Neurotoxic drug, (voltage sensitive) Na_v_ (1.1, 1.7, 1.5)- channel block	Decrease in slope, depolarization potential and action potential duration	10 µM	hiPSC-CM Cor4U^®^ mixture of ventricular, atrial, nodal cells	n/a	10 days after differentiation	Automated patch clamp	[26]
Thioridazine	Sedative, anti- psychotic drug, multi-channel block	Repolarization delays/arrhythmogenic effects	100 nM	hiPSC-CM iCell™ (mixture of ventricular, atrial, nodal cells)	n/a	32 days of differentiation +15–26 days	MEA	[87,88]
Tolterodine	Treatment of urinary incontinence, muscarinic receptor antagonist	clinical QT prolongation, early afterdepolarization	100–300 nM	hiPSC-CM iCell™ (mixture of ventricular, atrial, nodal cells)	n/a	32 days of differentiation +15–26 days	MEA	[87]
Vanoxerine	Serotonin-dopamine reuptake inhibitor	Clinical QT prolongation, multiple ion-channel effects, early afterdepolarizations	100 nM	hiPSC-CM iCell™ (mixture of ventricular, atrial, nodal cells)	n/a	32 days of differentiation +15–26 days	MEA	[87]
Vandetanib	Anti-cancer drug for thyroid gland, kinase inhibitor	Repolarization prolongation, arrhythmia like events	0.1–1 µM	hiPSC-CM iCell^2^™/Cor4U^®^ (mixture of ventricular, atrial, nodal cells)	n/a	32 days of differentiation n/a	MEA/VSO	[93]

**Table 4:** MEA based safety testing of drugs with cardiac indication using hiPSC-CM.

Substance	(Side of) Action	Effect	Min. Effective Conc.	Cell Type	Differentiation Protocol	Age/Maturation State	Platform	Reference
Amiodarone	Class III anti-arrhythmic drug, multi-channel block	Clinical QT prolongation	0.1–1 µM	hiPSC-CM iCell™ (mixture of ventricular, atrial, nodal cells)	n/a	32 days of differentiation +15–26 days	MEA	[87,88]
Azimilide	Class III anti-arrhythmic drug	FPDc prolongation, decrease in beating frequency, early after depolarization	0.3–1 µM	hiPSC-CM iCell™ (mixture of ventricular, atrial, nodal cells)	n/a	32 days of differentiation	MEA	[88]
Bay K 8644	Agonist of voltage sensitive dihydropyridine (DHP; L-Typ) Calcium channel	FPDc prolongation, decrease in beat frequency, positive inotropic	0.3–3 nM	hiPSC-CM iCell™ (mixture of ventricular, atrial, nodal cells)	n/a	32 days of differentiation +15–26 days	MEA	[87,88]
Bepridil	Class IV anti-arrhythmic drug, multi-channel block	Repolarization delays/arrhythmogenic effects	0.1–1 µM	hiPSC-CM iCell™/iCell^2^™/Cor4U^®^ (mixture of ventricular, atrial, nodal cells)	n/a	32 days of differentiation +15–26 days n/a	MEA	[87,88,93]
Dofetilide	Class III anti-arrhythmic drug, multi-channel block	Increase in FPD, TdP arrhythmias	3–100 nM	hiPSC-CM iCell™/iCell^2^™/Cor4U^®^ (mixture of ventricular, atrial, nodal cells) Pluricytes™	n/a	32 days of differentiation n/a n/a	MEA	[88,93,102]
E-4031	Class III anti-arrhythmic drug, hERG- channel block	prolonged FPD, severe arrhythmia in LQTS iPSC-CM	30–100 nM	hiPSC-CM iCell™/Cor4U^®^ (mixture of ventricular, atrial, nodal cells) GE Healthcare (Cytiva™), Stanford Cardiac Institute LQTS cells and control	n/a3D	32 days of differentiation +14–24 days n/a 30–60 days of differentiation +50 days	MEA	[85,97,98]
Flecainide	Class Ic anti-arrhythmic drug, multi-channel block	Decrease in spike amplitude, FPDc prolongation	1 µM	hiPSC-CM iCell™/Cor4U^®^ (mixture of ventricular, atrial, nodal cells) GE Healthcare (Cytiva™), Stanford Cardiac InstituteCPVT cells and control	n/a 2D/3D	32 days of differentiation +14–24 days n/a 20–30 days of beating	MEA Patch clamp	[85,98,103]
Ibutilide	Class III Anti-arrhythmic drug, multi-channel block	Arrhythmia like events, early after depolarizations	1–100 nM	hiPSC-CM iCell™/iCell^2^™/Cor4U^®^ (mixture of ventricular, atrial, nodal cells)	n/a	32 days of differentiation n/a	MEA/VSO	[88,93]
Ivabradin	Treatment of stable angina pectoris, If-channel inhibitor, heart rate reducing drug	Prolongation in APD, decrease in beating frequency	1 µM	Double reporter cell line, subtypes: ventricular, atrial, nodal, TBX5 Nkx2.5/hiPSC-CM	2D	35–40 days of differentiation	Patch clamp	[21]
JNJ303	IKv7.1- channel inhibitor	Small prolongation of FPDc	300 nM	hiPSC-CM iCell™/Cor4U^®^ (mixture of ventricular, atrial, nodal cells) GE Healthcare (Cytiva™), Stanford Cardiac Institute	n/a	32 days of differentiation n/a	MEA	[85]
Levocromakalim	Vasodilating drug, K_ATP_ opener	Membrane hyperpolarization, decrease in FPDc and beating frequency	1–3 µM	hiPSC-CM iCell™ (mixture of ventricular, atrial, nodal cells)	n/a	32 days of differentiation +15–26 days	MEA	[87,88]
Metoprolol	Anti- arrhythmic, anti- hypertonic drug, ß1-adreno receptor block	Induced arrhythmias, hERG block at higher concentrations	100 µM	hIPSC CM iCell^2^™/Cor4U^®^ (mixture of ventricular, atrial, nodal cells) CPVT cells and control	n/a 2D/3D	32 days of differentiation n/a 20–30 days of beating	MEA/VSO Patch clamp	[93,103]
Mexiletine	Class Ib anti-arrhythmic drug, Inhibiting Nav1.5- also hERG block	Reduce spike amplitude, cessation of spontaneous beating (100 µM)	1–10 µM,	hIPSC-CM iCell™/iCell^2^™ Cor4U^®^ (mixture of ventricular, atrial, nodal cells) GE Healthcare (Cytiva™), Stanford Cardiac Institute	n/a	32 days of differentiation +14–24 days n/a	MEA	[85,88,93,98]
Mibefradil	Treatment of angina pectoris and hypertension, multi-channel block	Shortening in FPDc, increase in beat frequency	0.3–1 µM	hiPSC-CM iCell™ (mixture of ventricular, atrial, nodal cells)	n/a	32 days of differentiation +15–26 days	MEA	[87,88]
Nifedipin	Vasodilating drug, I_CaL_ block	Shortening of FPDc, increase in beating rate	0.3–1 µM	hiPSC-CM iCell™/Cor4U^®^ (mixture of ventricular, atrial, nodal cells) GE Healthcare (Cytiva™), Stanford Cardiac Institute	n/a	32 days of differentiation +10 days n/a	MEA, automated patch clamp	[26,85,98]
NS- 1643	hERG-channel activator	Repolarization effect, decrease in FPDc, increase in beating frequency	3 µM	hiPSC-CM iCell™ (mixture of ventricular, atrial, nodal cells)	n/a	32 days of differentiation +15–26 days	MEA	[87,88]
Ouabain	Cardiac glycoside, Na^+^-K^+^- ATPase inhibitor	Repolarization effects, decrease in FPDc	10–100 nM	hiPSC-CM iCell™ (mixture of ventricular, atrial, nodal cells)	n/a	32 days of differentiation +15–26 days	MEA	[87,88]
Propranolol	Class II anti-arrhythmic drug, beta- receptor block	Early afterdepolarization, decrease in beating frequency	10 µM	hiPSC-CM iCell™ (mixture of ventricular, atrial, nodal cells)	n/a	32 days of differentiation	MEA	[88]
Quinidine	Class Ia anti-arrhythmic drug, multi-channel block (Nav1.5, Cav1.2, hERG)	FPDc prolongation, reduced spike amplitude, repolarization delays/arrhythmogenic effects	0.3–10 µM	hiPSC-CM iCell™/iCell^2^™ Cor4U^®^ (mixture of ventricular, atrial, nodal cells) GE Healthcare (Cytiva™), Stanford Cardiac Institute Fibroblast-derived iPSC-CM (67% ventricular, 5% nodal, 28% atrial)	n/a 3D	32 days of differentiation +15–26 days 65–95 days after differentiation induction	MEA, Low impedance MEA	[85,87,88,93,98,104]
Ranolazine	Angina pectoris treatment, multichannel (Na and hERG block)	FPDc prolongation, clinical QT prolongation, repolarization prolongation	0.3 µM, clinical conc. <100 µM	hiPSC-CM iCell iCell^2^™ Cor4U^®^ (mixture of ventricular, atrial, nodal cells) GE Healthcare (Cytiva™), Stanford Cardiac Institute		32 days of differentiation +15–26 days (14–24 days) n/a	MEA	[85,87,88,93]
Sotalol	Anti-arrhythmic drug, beta adreno receptor block	Repolarization prolongation, arrhythmogenic effects, hERG- channel block	15 µM	hiPSC-CMs iCell^2^™/Cor4U^®^ (mixture of ventricular, atrial, nodal cells) Fibroblast-derived iPSC-CM (67% ventricular,5% nodal, 28% atrial)	n/a 3D	32 days of differentiation n/a 65–95 days after differentiation induction	Low impedance MEA	[93,104]
Verapamil	Class VI anti-arrhythmic drug, inhibits hERG, I_Cal_-typ calcium channels, Multi-channel block	Shortening of FPDc, increase in spontaneous beat rate; shortening in APD_20_ and APD_90_	0.1–0.3 µM; 1 µM	hiPSC-CM iCell™ (mixture of ventricular, atrial, nodal cells) Pluricytes™	n/a	32 days of differentiation n/a	MEA; patch clamp	[98,102]
Vernakalant	Class III anti-arrhythmic drug, used for cardioversion of atrial fibrillation, atrial potassium- channel block	APD prolongation, partly arrhythmogenic effects,	-	Double reporter cell line, different subtypes (ventricular phenotype)	2D	20–30 days post induction of differentiation	Patch clamp	[21]
ZD 7288	Selective hyperpolarization-activated cyclic nucleotide-gated channel blocker, I_f-_ current inhibitor	Negative chronotropic effect, FPDc prolongation	3–30 nM	hiPSC-CM iCell™ (mixture of ventricular, atrial, nodal cells)	n/a	32 days of differentiation +15–26 days	MEA	[87,88]

## 5. Disease Modeling Using hiPSC-CM

Cardiovascular diseases (CVD) are the number one cause of death globally according to the WHO. This group of disease is not only present in developed countries but also in low income countries. CVD affects both the blood vessels and the heart and range from coronary heart disease, thrombosis, over structural abnormalities of the heart and arrhythmias to stroke and heart failure [83].

In order to understand the pathological mechanisms of the disease iPSC-derived cardiomyocytes generated from patient cells like dermal fibroblasts or blood cells showed high potential as a tool to not only generate immortalized cell lines from healthy donors but also from diseased patients [105]. The generated cell lines can show disease-specific phenotypes [99]. Especially physiological characteristics can be determined when carrying the mutations in the cardiac relevant genes. This suggests that the disease phenotype can be recapitulated in vitro. To further analyze the pathogenic mechanisms causing the disease and the disease specific phenotype patient originated iPSC-CM were analyzed and afterwards genetically fixed. Here, RNA interference can be used for silencing or suppressing mutant genes [106]. To determine and control the effects caused by mutations the initial genetic defect was induced and replicated in hESC derived CM on the contrary. A correction of the defect led to a normalization of the analyzed parameter whereas the induction led to the diseased phenotype [107]. Besides a better understanding of heart disease, the transgenic models should contribute to testing of cardiogenic drugs on their positive therapeutic as well as detrimental side effects. Moreover, the sensitivity of patients for side effects of drugs can be evaluated and, the clinical vulnerability of high-risk groups (population) to drug induced-cardiotoxicity can be set into consideration. This testing of therapies *in vitro* is another promising tool [99,108].

**Figure 3:**
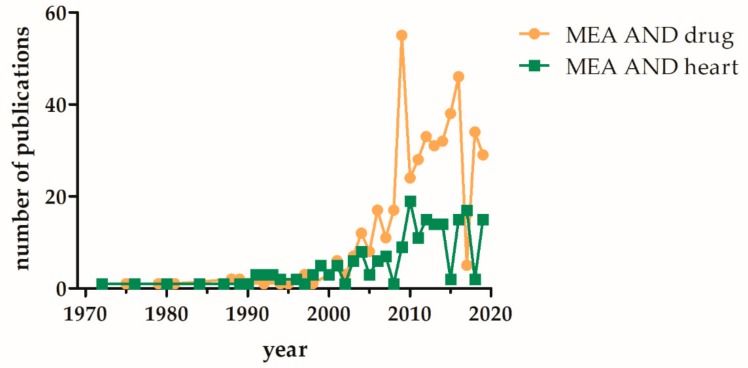
Increase of PubMed listed publications involving MEA based analysis of “heart” or “drugs” over the last five decades. The terms (multielectrode array and drug) or (microelectrode array and drug) and (multielectrode array and heart) or (microelectrode array and heart) were used for the PubMed search (date: Sept 2019).

For the analysis and validation of the generated iPSC-CM based disease models patch clamp analyses are still the gold standard, despite the number of MEA based measurements in increasing (Figure 3). In this part we want to give a selective overview about already developed disease models for cardiac disease generated from human iPSCs, where the usage of MEA platform is additionally mentioned. 

## 6. Overview of Developed Disease Models

Ping Liang et al. generated a library of iPSC-derived CM from patients suffering from various hereditary cardiac disorders to show that cardiac drug toxicity differs between different pathophysiological conditions. The iPSC-CM was generated from patients with hereditary long-QT syndrome, familial hypertrophic cardiomyopathy and familial dilated cardiomyopathy. They have shown that patients that already suffer from a heart disease have a higher incidence to show adverse effects arising from their medical treatment. They seem to have a higher sensitivity to cardiotropic drugs and can have a higher risk for arrhythmias, which possibly are leading to death [99].

In 2014 Zhang et al. generated cardiomyocytes derived from iPSC from patients with recessive, life-threatening cardiac arrhythmia of Jarvell and Lang–Nielsen syndrome. They gave new insights into the pathological mechanisms and showed enhanced sensitivity to proarrhythmic drugs in the generated cell-based disease model using MEA technology and patch clamp [109].

Considering the literature of the last years for ion channelopathies, these appear to be in focus of disease modeling, revealing many well-established human iPSC generated disease models. Among these the long QT syndrome is the most common. The first model has been developed by Moretti et al. (2010). Ventricular and atrial cells in contrast to nodal type or healthy control cells, have shown significantly increased APDs [110]. The response of sporadic Long QT1-iPSC-CM to small molecule inhibitors has been analyzed measuring changes in the FPD with the MEA platform [97].

A mutation in the gene of the sodium voltage-gated channel (Nav1.5) alpha subunit 5 (SCN5A) for example is leading to conduction defects, phenotypes of the LQT3 and Brugada syndrome due to a gain and loss of function [24]. A review on modeling long QT syndrome with the aid of iPSC-CM can be found by Sala et al. [111].

Another channelopathy that has been used for the generation of a disease model is the catecholaminergic polymorphic ventricular tachycardia (CPVT). An incorrect and insufficient Ca^2+^ handling (inclusive spontaneous release or sequestration) is leading to this adrenergically mediated polymorphic ventricular tachycardia [112]. Sasaki et al. generated CM from CPVT patient- derived iPSCs and identified S107 as a potential therapeutic agent since a pre-incubation with S107 led to a reduction of isoprenaline induced delayed afterdepolarizations [113]. Acimovic et al. (2018) developed a CPVT model using a novel ryanodine receptor mutation and further analyzed the response to a treatment with flecainide and metoprolol [103].

Furthermore, models of structural myopathies have been developed: among these, the hypertrophic cardiomyopathy (HCM) and the familial dilated cardiomyopathy (DCM) are the most common being analyzed. HCM associated death is mostly caused by ventricular fibrillation developed from ventricular arrhythmias. Despite that not all pathophysiological mechanisms are known yet, some detected mutations have been the basis of developed disease models. HCM iPSC derived cardiomyocytes have shown enhanced sarcomere arrangement [114], deviating electromechanical properties such as delayed after depolarizations and calcium handling [115]. DCM is leading to systolic dysfunction such as decreased ejection fraction due to an expanded size of the left ventricle combined with decreased chamber thickness. Several known mutations have been fundamental for the generation of a DCM model, including *MYH7* [116], *TNNT* [117], *LMNA* (laminin A/c) [118], Desmin [119], Titin [120] and RBM20 RNA-binding motif protein 20 [121,122]. On the cellular level an increase in cell size, abnormal sarcomere structure and organization e.g., sarcomeric α actinin and defective calcium handling (altering calcium machinery) [121] could be seen within these models. For further detailed and completely information we would recommend the review by Giacomelli et al. [123].

Moreover, cardiomyocytes have been generated from patients with Duchenne muscular dystrophy iPSCs showing phenotypical deficiency of dystrophin and increased Ca levels in resting cells. The before mentioned disease caused by a high quantity of mutations is characterized by a knockout of the dystrophin protein resulting in muscle degeneration [124]. For further detailed reading we recommend the systematic review on cardiomyopathy phenotypes in iPSC-CM (HCM and DCM) by Eschenhagen and Carrier [125].

Besides the generation of disease models from patient derived cells, new gene editing technologies find application: de la Roche et al. generated a model for the Brugada syndrome carrying the A735V mutation in the *SCN5A* gene introduced by CRISPR/Cas9 in order to be independent of the patient’s genetic background. The generated CM showed electrophysiological alteration such as decreased upstroke velocity and sodium current density [126].

Among the current literature the number of developed models that are not primarily heart diseases (structural caused heart disease) but show heart related symptoms is increasing. For example a model for Chagas disease, a parasite caused infection by *Trypanosoma cruzi* that is associated with cardiomyopathy symptoms since the parasites are replicating in the cardiomyocytes, was developed by Bozzi et al. after infection of iPSC-CM with *Trypanosoma cruzi* [127]. Lee et al. developed a model for autosomal dominant polycystic kidney disease (ADPKD) form patient derived iPSC-CM showing mutations in the *PKD1*/*PKD2* gene [128], to name only two of them.

Since the generation of disease models with patient derived iPSC-CM was developed and practiced within the last years, the first scientific outcome has been produced in a more detailed understanding of the pathophysiological mechanisms that underlie known symptoms. Several recent studies were performed:

Using patient derived iPSC-CM from Fabry disease combined with gene editing technology Birket et al. analyzed the functional consequences of underlying genetic defects. They have shown the accumulation of GL-3 and alterations in excitability and calcium handling in cardiomyocytes. Moreover LIMP-2, shown to accumulate in the cells, was detected as a new potential biomarker [129].

Caluori et al. developed a system combining MEA platform with cantilever of atomic force microscopy (AFM) in order to analyze the topography, beating force and electric events simultaneously of control and DCM-derived iPSC-CM. Meanwhile substances for the cell characterization and toxicity testing have been administrated [130]. 

Moreover, microtissues have been generated from the disease model cardiomyocytes for hypertrophic cardiomyopathy HCM [131] and for catecholaminergic polymorphic ventricular tachycardia CPVT [132]. The HCM model was used to analyze contraction force development and calcium transient under mechanical overload revealing that both genetic defects and environmental stress reinforce dysfunctions in contractility [131]. The CPVT tissue construct examined molecular and cellular abnormalities and is confirming the approach of generating tissue like structures for the analysis of arrhythmia, which is mostly generated by the connection of many cells [132]. 

Another approach for the further use of these disease models and an improved reliability of the results/predictions are large scale simulations in in silico models. In order to enable this approach for the LQT syndrome with regards to the phenotypical variation (due to the large amount of different mutations) this system was developed and showed comparable results to experimental data concerning electrophysiological properties. Additionally, this simulation can facilitate the understanding of further biophysical mechanisms [133].

## 7. Conclusions and Outlook

Cardiomyocytes generated from human iPSCs are used to study heart development, cardiac function and heart diseases, and to develop novel pharmacological therapeutics [11,21,23,134]. This involves a detailed electrophysiological characterization of these cardiomyocytes under physiological and pathological conditions. Due to its non-invasive, label-free character MEA methodology has become a widely used tool to assess the electrical properties of cells in vitro. Besides iPSC-derived cardiomyocytes, acquisition of the FP by MEA assays can also be applied to any other cell type that shows electrical activity, including adult cardiomyocytes or neuronal cells. However, MEA measurements are not only restricted to cell monolayers, but can also be performed on slices of cardiac and brain tissue to better simulate in vivo conditions. Similarly, the replication of human physiology can be enhanced when MEA technology is combined with in vitro generated organoids. Therefore, MEA platforms can become a valuable tool in organ-on-a-chip engineering, promoting the clinical translation of acquired data [135,136].

As a high-throughput technology, MEA devices are particular important for the identification of novel therapeutics in drug research. The combination of MEA systems and iPSCs has been successfully applied in pharmacological studies and disease modeling, suggesting iPSC-based MEA measurements as a powerful approach for the development of personalized therapies that allow a more specific therapeutic intervention compared to conventional treatment options [137,138,139]. In this concept, patient-derived iPSCs are obtained by reprogramming of fibroblasts and induced to differentiate into cardiomyocytes. Subsequent analyses, e.g., transcriptomics and proteomics, enable the identification of molecular targets needed for pharmacological therapy. In a second step, the potency and efficiency of tested drugs is evaluated by MEA measurements, which in turn can provide important information to establish personalized drug treatments. Thus, MEA technology will help to open a new gateway for novel therapies in cardiovascular diseases. 

## Figures and Tables

**Table 1:** Functional parameters acquired by FP measurements using MEA Systems.

FP Morphology	Physiological Parameter
Spatiotemporal Assessment	Propagation velocity, direction origin of AP spread
FP Duration	QT interval of AP
FPs Over Time	Beating frequency
Spike Amplitude	Na^+^ current
Spike Plateau	Ca^2+^/K^+^ current
Beat-to-Beat Interval	AP duration

**Table 2:** List of CiPA compounds defined by CiPA initiative * (May 2016) [86].

High TdP Risk	Intermediate TdP Risk	No or Very Low TdP Risk
	Astemizole	
	Chlorpromazine	Diltiazem
Azimilide	Cisapride	Loratadine
Bepridil	Clarithromycin	Metoprolol
Dofetilide	Clozapine	Mexiletine
Ibutilide	Domperidone	Nifedipine
Quinidine	Droperidol	Nitrendipine
Vandetanib	Terfenadine	Ranolazine
Disopyramide	Pimozide	Tamoxifen
D,l Sotalol	Risperidone	Verapamil
	Ondansetron	

## Supplementary Materials

The supplementary materials are available online at https://www.mdpi.com/2073-4409/8/11/1331/s1.

## Abbreviations

2D	Two-dimensional
ADPKD	Autosomal dominant polycystic kidney disease
AFM	Atomic force microscopy
AP	Action potential
APD	Action potential duration
ATM	Atomic force microscopy
AV Node	Atrioventricular node
HCM	Hypertrophic cardiomyopathy
Ca^2+^	Calcium
CiPA	Comprehensive in vitro proarrhythmia assay
CM	Cardiomyocytes
CPVT	Catecholaminergic polymorphic ventricular tachycardia
CSAHI	Consortium for Safety Assessment using Human iPS cells
CVD	Cardiovascular disease
DCM	Familial dilated cardiomyopathy
FP	Field potential
FPDc	Field potential duration (corrected)
FRET	Fluorescence resonance energy transfer
GL-3	Basic helix-loop-helix proteins, GLABRA3
gpAPD	Guinea pig papillary muscle action potential assay
hERG	Human ether-a-go-go Related gene-voltage sensitive potassium channel
hiPSC	Human induced pluripotent stem cell
K^+^	Potassium
LIMP-2	Lysosomal integral membrane protein 2
LMNA	Lamin A/C
MEA	Multi-/micro-electrode array
MYH7	Myosin heavy chain 7
Na^+^	Sodium
PKD1/2	Polycystic kidney disease 1
RNA	Ribonucleic acid
SCN5A	Sodium voltage-gated channel alpha subunit 5
TBX5	T-box transcription factor
TdP	Torsade-de-Pointes-Tachycardia
TnnT	TroponinT
VSO	Voltage-sensitive optical system
WT	Wild type
